# Twenty years of courage and compassion: Leading palliative care with dignity and vision

**DOI:** 10.1017/S1478951526102284

**Published:** 2026-04-14

**Authors:** Isabel Galriça Neto

**Affiliations:** 1 Palliative Care Department, Palliative Care Unit Hospital Luz-Lisboa, Lisboa, Portugal; 2 Faculdade de Medicina, Universidade de Lisboa, Lisboa, Portugal; 3 Faculdade de Medicina, Universidade Católica Portuguesa, Lisboa, Portugal

This essay reflects on 2 very special decades of my life.

After more than 30 years working in palliative care (PC) and 20 of them as Head of the PC Unit in a Lisbon private hospital for acute care, the moment of farewell inevitably arrives. It is not a farewell from a place, nor from a cause, but from a position of formal leadership.

Gordon Brown (2007) wrote a book named “Courage: Portraits of Bravery in the Service of Great Causes” and Dame Cicely Saunders was one of the examples he described. I firmly agree with him, and I thank the inspiration of such a great woman.

When I began this journey in 2006 invited by the administration, it required vision, courage, and a bit of boldness. Establishing and consolidating the first PC unit within the biggest acute private Portuguese hospital setting demanded precisely that: courage to challenge dominant paradigms – the dominant model of acute care, even for fragile and vulnerable patients – courage and vision to defend the value of care that does not aim to cure, courage to insist that dignity remains central even when technology and urgency dominate the corridors, and boldness when we almost ignored the dimension of obstacles that were to come.

As a PC team, we walked, as it were, on the shoulders of giants, our patients, and their families. They were the reason for our path, the true teachers of our discipline. Every clinical decision, every organizational change, and every late-night discussion were anchored in their stories. They entrusted us with their vulnerability at the most fragile moments of their lives. It is to them that this farewell is ultimately dedicated.

## Building a model grounded in ethics and PC values

What was built over these 20 years? Where was the model of care grounded?

From the beginning, the PC approach was founded on a clear ethical framework: respect for autonomy, relief of suffering, proportionality in therapeutic interventions, and unwavering commitment to human dignity. Our ethical stance was not theoretical; it was practical. In a hospital environment often driven by protocols, targets, and technological innovation, we insisted that the person – not the disease – must remain at the center. For us, humanization is not an accessory, it is the core of excellent care.

I drew inspiration from pioneers of the field, like Robert Twycross, and my prior experience in Oxford, Madrid, and Canada. I have relied on the recommended values for the practice of PC and developed a structured model: based on promoting comfort – not prolonging life at any cost – interdisciplinary, holistic, and integrated care within the hospital system. Physicians, nurses, psychologists, social workers, chaplains, and therapists worked side by side. The complexity of suffering, in all its dimensions, demands collective intelligence and shared responsibility. True interdisciplinarity was one of our main pillars and innovations, as it was the inclusion of families as our area of care.

I also reflected deeply on the structural challenges described by leaders such as Eduardo Bruera (2024), who warned about the systemic obstacles to the development of PC: insufficient training, inadequate funding, cultural resistance, and the persistent misconception that PC might equal abandonment.

## Results, challenges, and added value

Over the past 20 years, as a PC team, we have achieved both measurable and immeasurable results. We have treated more than 11,000 patients and their families, over 70% of whom had a Palliative Performance Scale (PPS) < 40%. About 55% of these patients were oncological, while the remainder were non-oncological. The discharge rate ranged between 45% and 57%.

We have been an ESMO-Designated Centre of Integrated Oncology and PC since 2011 and we were included in the Joint Commission International accreditation of our hospital since 2018. We trained more than 100 professionals, implemented protocols, improved symptom control, reduced inappropriate interventions at the end of life, and fostered a culture of dialogue around goals of care. We strengthened collaboration with other departments and built bridges between hospital and community services.

In doing so, we added value – not only human value but also systemic value. The work of organizations such as Center to Advance Palliative Care (CAPC) (2025) has demonstrated that PC improves quality of life and patient satisfaction, reduces the burden of symptoms, and optimizes resource use while reducing costs. This is truly value-based health care. I experienced this reality firsthand: better symptom control reduced emergency visits; earlier conversations about care goals prevented futile and burdensome interventions.

I was also mindful of the broader debates on evident PC benefits (Gomes 2015) and on health expenditure and resource allocation. In many systems, and despite the evidence, a disproportionate share of healthcare spending keeps occurring in the last months of life, often without corresponding benefit. Thought leaders such as Michael Porter and Sir Muir Gray have highlighted the need to realign systems toward value-based, patient-centered care. Our unit sought to embody that realignment in daily practice.

Yet the path was never without challenges. There were moments of group resistance, times of limited staffing, and the ever-present tension between industrialized healthcare processes and individualized care. I witnessed the rise of new therapies, increasingly sophisticated technologies, and growing pressure for rapid throughput. Each innovation brought opportunities but also risks.

## The present moment: Risks and opportunities

We are in an era marked by the industrialization of health care – which leaves less room for holistic care – rapid biomedical innovation, and digital transformation. Artificial intelligence, personalized medicine, and advanced oncological treatments all promise unprecedented possibilities, many of which truly represent progress.

However, not every novelty is an authentic advance. Some innovations may extend life without necessarily enhancing its quality. Some technologies risk distancing professionals from patients, replacing attentive listening with screen-focused interaction.

Development in PC has never been linear. Progress requires institutional commitment, education, and research. It also requires cultural change – both within the medical profession and in society at large, including death in our lives and not looking at it as defeat.

The notable Portuguese neurosurgeon João Lobo Antunes reminded us (Lobo Antunes 2015) the irreducible complexity of human suffering: “I don’t know what awaits us, but I do know what worries me: it is that medicine, excited by science, seduced by technology and stunned by bureaucracy, will erase its human face and ignore the unique individuality of those who suffer, because although more and more ways of treating are being invented, the way to alleviate suffering without empathy or compassion has not yet been discovered.”

We must guard against the erosion of presence. We must ensure that efficiency does not suffocate empathy. The future of PC depends not only on scientific development but also on preserving its identity – its commitment to accepting death while enhancing life, concerned with healing not curing, and promoting patient-centered rather than disease-centered care.

## Learning from mistakes and defending good practice

Over 2 decades, I also observed examples that the principles and practice of PC are not yet fully known by many professionals, and sometimes, well-intentioned interventions caused avoidable suffering.

These experiences reinforced our conviction that PC must be integrated early, not reserved for the final weeks or days. It must be a philosophy embedded across services practices, not an isolated specialty called only when “nothing more can be done.” In truth, there is always something to be done to provide more efficient and humanized care.

## Continuing the journey

As I step down from leadership, I do so with serenity and gratitude. It was all worth it! Leadership is temporary; the mission endures. I leave the position, not the cause. I remain a citizen, a professional, and an advocate for a healthcare system that honors the most vulnerable.

The future demands that we in PC continue building on solid foundations: PC must be both rigorous and compassionate. We must persevere – ensuring academic rigor, expanding training programs, promoting research, and strengthening collaboration between hospital and community.

Above all, PC identity must be preserved. A discipline that stagnates fades away. However, a discipline that sacrifices its core values in pursuit of novelty loses its soul. True innovation in PC lies not only in new pharmacological agents or digital tools but in deeper communication skills, earlier integration, and broader societal understanding.

## Gratitude and hope

To my teachers and masters, for what they generously passed on to me.

To my family and friends, and mainly GOD, the great pillar of my life.

To the Team with whom I shared these 20 years: thank you. No head of department accomplishes anything alone. Each success was collective. Each difficulty was carried together. The bonds formed through shared responsibility for suffering are unlike any others.

To the patients and families who trusted us: you shaped us more than any textbook ever could. You were our compass.

To the institution and administration: thank you for the space to build, to argue, to innovate, and sometimes to resist.

As I say goodbye to this chapter, I do so not with nostalgia but with hope. And as Bill Breitbart says (2025), “Hope is the Courage to Create an Uncertain Future.” The next generation will bring new ideas, new energy, and new perspectives. May they be bold enough to innovate and wise enough to preserve what is essential.

And so, I conclude not with an ending but with an open horizon, as the unique Buzz Lightyear points: to infinity and beyond!
